# Seroprevalence of Hepatitis B Virus among Adults at High Risk for HIV Transmission Two Decades after Implementation of Nationwide Hepatitis B Virus Vaccination Program in Taiwan

**DOI:** 10.1371/journal.pone.0090194

**Published:** 2014-02-26

**Authors:** Hsin-Yun Sun, Chien-Yu Cheng, Nan-Yao Lee, Chia-Jui Yang, Shiou-Haur Liang, Mao-Song Tsai, Wen-Chien Ko, Wen-Chun Liu, Pei-Ying Wu, Cheng-Hsin Wu, Hsi-Hsun Lin, Chien-Ching Hung

**Affiliations:** 1 Department of Internal Medicine, National Taiwan University Hospital and National Taiwan University College of Medicine, Taipei, Taiwan; 2 Department of Internal Medicine, Tao-Yuan General Hospital, Ministry of Health and Welfare, Tao-Yuan, Taiwan; 3 Department of Internal Medicine, National Cheng Kung University Hospital and College of Medicine, Tainan, Taiwan; 4 Department of Internal Medicine, Far Eastern Memorial Hospital, New Taipei City, Taiwan; 5 Department of Medicine, E-Da Hospital/I-Shou University, Kaohsiung, Taiwan; 6 Center for Infection Control, National Taiwan University Hospital, Taipei, Taiwan; 7 Department of Medical Research, China Medical University Hospital, Taichung, Taiwan; 8 China Medical University, Taichung, Taiwan; Centers for Disease Control and Prevention, United States of America

## Abstract

**Background:**

Seroprevalence of hepatitis B virus (HBV) after implementation of universal neonatal HBV vaccination and catch-up vaccination programs remains rarely investigated among the adults who were born in the vaccination era (in or after 1986) and engaged in high-risk sexual behaviors.

**Materials and Methods:**

Between 2006 and 2012, we determined HBV surface antigen ([HBsAg), anti-HBs, and HBV core antibody (anti-HBc), hepatitis C virus antibody (anti-HCV) and rapid plasma reagin titers among HIV-infected men who have sex with men (MSM) born during 1984–1985 (Group I: 244 persons) and those born in or after 1986 (Group II: 523), and HIV-uninfected MSM (Group III: 377) and heterosexuals (Group IV: 217) born in or after 1986. Prevalence and incidence of HBV infection were estimated and multivariate analysis was performed to identify factors associated with HBsAg positivity.

**Results:**

Compared with Group I, Groups II-IV had a significantly lower prevalence of HBsAg positivity (7.8% vs 3.7%, 2.4%, and 3.2%, respectively); and the prevalence of anti-HBc positivity was also lower for Groups III and IV (30.3% vs. 19.6%, and 18.0%, respectively), but no difference was observed between Groups I and II (30.3% vs. 26.3%). In multivariate analysis, HBsAg positivity was significantly associated with syphilis (adjusted odds ratio, 2.990; 95% confidence interval, 1.502–5.953) and anti-HCV positivity (adjusted odds ratio, 3.402; 95% confidence interval, 1.091–10.614). In subjects of Group II with all-negative HBV markers at baseline, the incidence rate of HBsAg seroconversion was 0.486 episodes per 100 person-years; and for those who received combination antiretroviral therapy containing lamivudine and/or tenofovir, none developed HBsAg seroconversion during the follow-up.

**Conclusions:**

Among the adults who were born in or after 1986 and engaged in high-risk sexual behaviors in Taiwan, neonatal HBV vaccination and catch-up vaccination programs conferred long-term protection against HBsAg seroconversion and HBsAg positivity was associated with syphilis and anti-HCV positivity.

## Introduction

Hepatitis B virus (HBV) vaccination with high immunogenicity of 90 to 95% in healthy adults aged less than 40 years has effectively decreased the incidence of acute HBV infection [1]. In areas that implement universal neonatal HBV vaccination program such as Taiwan and Alaska, the incidence of acute HBV infection [2], [3], prevalence of chronic HBV infection [3], [4], and incidence of hepatocellular carcinoma in children and the mortality of chronic liver disease as well as hepatocellular carcinoma in persons aged 5–29 years have significantly declined [3], [5], [6].

Despite a low incidence, cases of HBV infection among adults who had undergone HBV vaccination at birth in Alaska and Taiwan have been reported [2], [7], [8]. In Alaska, a 15-year follow-up study after implementation of HBV vaccination program showed that the definite breakthrough HBV infections occurred at an incidence of 0.61 per 1000 persons per year (crude incidence, 1.2% [10/835]) in subjects who had initial response to vaccination [7]. In Taiwan, the incidence of acute HBV infection among the individuals aged 15–24 years who were born in the era of nationwide HBV vaccination was 0.51 per 100,000 population, while the incidence was 2.28 per 100,000 population in unvaccinated cohort [2].

Given the shared transmission routes of HIV and HBV, co-infection with HBV and HIV is common. It has been shown that seropositivity for syphilis and HIV infection, and the number of lifetime sexual partners are associated with increased risk of HBV infection [9]; furthermore, sexual behavior, the number of life-time sexual partners, and receptive anal intercourse were significantly associated with HBV infection in men who have sex with men (MSM) [10], [11]. Compared with HIV-uninfected individuals, HIV-infected patients are less likely to clear HBV in the presence of acute HBV infection [12], and are at increased risk of development of chronic infection [13], cirrhosis of the liver, and end-stage liver disease [14]. Thus, routine screening for HBV and vaccination are highly recommended for all HIV-infected adults to prevent primary HBV infection [15]. However, compared with HIV-uninfected individuals, HIV-infected patients have a lower serological response [16], lower titer of protective antibody [17], and faster rate of antibody decline after HBV vaccination.

In this study, we aimed to investigate whether neonatal HBV vaccination and catch-up vaccination could confer long-term protection against HBV infection among HIV-infected patients and adults at risk for HIV infection; to identify factors associated with HBV infection; and to estimate the incidence of HBV surface antigen (HBsAg) seroconversion among HIV-infected patients in Taiwan where the background prevalence of HBsAg positivity was estimated 13–25% among the unvaccinated adults [18].

## Materials and Methods

### Setting and Study population

Nationwide HBV vaccination program in Taiwan was initially implemented to vaccinate newborns of HBsAg-positive mothers alone with administration of immunoglobulin to infants born to mothers who tested positive for HBV envelope antigen (HBeAg) in July 1984, which subsequently extended to cover all newborns after July 1986 [19] and susceptible preschool children, school children, teenagers, and then to adults from July 1987 to 1990. Since 1991, the vaccination records of school children aged 7 years were checked, and those children who were unvaccinated or incompletely vaccinated would be given catch-up HBV vaccination [19], [20]. The coverage rate of the nationwide HBV vaccination program was estimated to be 86.9–98.0% [20]. Thus, in this study, persons who were born in or after 1986 were assumed to have undergone HBV vaccination at birth or in the catch-up vaccination programs.

In April 2006, an expanded program of free-of-charge, anonymous counseling and testing (VCT) for HIV infection for persons who considered themselves at risk for HIV infection and related sexually transmitted diseases (STDs) was implemented at the National Taiwan University Hospital [21]. In addition to HIV testing, serological tests for *Treponema pallidum*, HBV (HBsAg, anti-HBs antibody, anti-HBV core antibody [anti-HBc]), and hepatitis C virus (HCV) were also provided. An anonymous, self-administered questionnaire interview was performed to obtain demographics, and information on sexual practices, risk behaviors for HIV infection and STDs. VCT clients gave written informed consent that was signed using a code consisting of birth year and the initial alphabet and the last four digits of identification card number [21]. For HIV-infected patients, those serological tests were performed at baseline and at least annually during the follow-up for those who were negative for all serological markers by following the national HIV treatment guidelines [22]; HBV vaccination was suggested to those who were negative for the three HBV markers.

From April 2006 to December 2012, we enrolled individuals born in or after 1986 who sought VCT or HIV care, and those born during 1984–1985 who sought HIV care at this university hospital and four other major designated hospitals in Taiwan (three in northern and two southern Taiwan). Those born in or after 1986 consisted of three groups: HIV-infected MSM (Group II), HIV-uninfected MSM (Group III), and HIV-uninfected heterosexuals (Group IV), while HIV-infected MSM born during 1984–1985 (Group I) were comparators. The incidence of HBsAg seroconversion was assessed in HIV-infected patients with all-negative HBV markers at baseline. The date of HBsAg seroconversion was arbitrarily defined as the mid-point between the date of the last negative and that of the first positive HBsAg results. The data were censored on the last sampling date for determination of HBV serostatus if the results of HBsAg remained negative. National Taiwan University Hospital Research Ethics Committee approved the study and waived for the need of informed consent.

### Data collection

A computerized data collection form was used to retrieve the demographic and clinical data of all subjects, which included birth year, gender, risk behaviors, and test results of anti-HCV antibody, HBsAg, anti-HBs antibody, and anti-HBc antibody at baseline; and data of baseline CD4+ lymphocyte count and plasma HIV RNA load of HIV-infected patients.

### Laboratory investigations

HBsAg, anti-HBs antibody, and anti-HBc antibody were determined with the use of enzyme immunoassay (Abbott Laboratories, Abbott Park, IL). Antibodies to HCV were determined with the use of a third-generation enzyme immunoassay (Ax SYM HCV III, Abbott Laboratories, North Chicago, IL). Plasma HIV RNA load were quantified using RT-PCR (Roche Amplicor, version 1.5, NJ) with a lower detection limit of 400 (2.60 log_10_) copies/mL, and CD4 counts were determined using FACFlow (BD FACS Calibur, Becton Dickinson, CA). The diagnosis of syphilis was made on the basis of a titer of rapid plasma reagin (RPR) ≧1:4 (RPR Card test; Becton-Dickinson) and confirmed by *T. pallidum* particle agglutination (SERODIA-TPPA; Fujirebio Taiwan Inc., Taoyuan, Taiwan) assay on the basis of a titer of ≧1:320 [23]. HIV infection was diagnosed by detection of anti-HIV-antibody using enzyme-linked immunosorbent assay or particle agglutination and confirmed by Western Blot test. Anti-HBs titers of 10 mIU/mL or more were regarded as positive and protective against HBV infection.

### Definitions

All-negative HBV markers were defined as HBsAg (−)/anti-HBs (−)/anti-HBc (−); serological markers for immunity against HBV after vaccination as HBsAg (−)/anti-HBs (+)/anti-HBc (−); isolated anti-HBc pattern as HBsAg (−)/anti-HBs (−)/anti-HBc (+); HBsAg carriage as HBsAg (+)/anti-HBs (−)/anti-HBc (+); and natural infections as anti-HBc (+) regardless of status of HBsAg and anti-HBs.

### Statistical analysis

All statistical analyses were performed using SPSS software version 17.0 (SPSS Inc., Chicago, IL). Categorical variables were compared using χ^2^ or Fisher's exact test whereas non-categorical variables were compared using Mann-Whitney U test. A multivariable logistic model was used to estimate the effects of multiple variables on the HBsAg positivity. The incidence rate of HBV seroconversion during the study period was calculated as the number of episodes of HBV seroconversion per 100 persons-years of follow-up (100 PYFU). All tests were two-tailed and a *P* value <0.05 was considered statistically significant. HIV-infected patients were followed until the end date of the study (June 30, 2013), death before June 30, 2013, and the last date of follow-up for those who were lost to follow-up, whichever occurred first.

## Results

### Characteristics of the four groups of subjects

During the 6.5-year study period, 244 HIV-infected MSM who were born during 1984–1985 (Group I), and 523 HIV-infected MSM (Group II), 377 HIV-uninfected MSM (Group III), and 217 HIV-uninfected heterosexuals (Group IV) who were born in or after 1986 were included (Table 1). For those born in or after 1986, a significantly higher proportion of HIV-infected MSM (Group II) had syphilis than HIV-uninfected MSM (Group III) or heterosexuals (Group IV) (*P*<0.001), while HIV-infected MSM born during 1984–1985 (Group I) were more likely to have syphilis than those born in or after 1986 (Group II-IV) (*P*<0.001) (Table 1). Likewise, for those born in or after 1986, HIV-infected MSM (Group II) had a higher prevalence of anti-HCV positivity than HIV-uninfected MSM (Group III) or heterosexuals (Group IV) (*P* = 0.045), while the prevalence was similar between the HIV-infected patients born during 1984–1985 (Group I) and those born in or after 1986 (Group II) (*P* = 0.570).

**Table 1:** Clinical characteristics of HIV-infected men who have sex with men (MSM) who were born during 1984–1985, HIV-infected MSM born in or after 1986, HIV-negative MSM born in or after 1986, and HIV-negative heterosexuals born in or after 1986.

	Group I: HIV- infected MSM, 1984–1985	Group II: HIV- infected MSM, 1986	Group III: HIV- uninfected MSM, 1986	Group IV: HIV-uninfected heterosexuals, 1986	*P* a value	*P* b value	*P* c value
Case no.	244	523	377	217	NA	NA	NA
Age, mean (SD), year	28.5 (0.5)	24.8 (1.9)	25.6 (1.4)	25.4 (1.6)	<0.001	<0.001	<0.001
HIV infection							
CD4 count, mean (SD), cells/μL	370.4 (215.8)	401.9 (276.5)	NA	NA	NA	0.197	NA
CD4<200 cells/μL, % (n)	21.7 (39/180)	16.7 (46/275)	NA	NA	NA	0.186	NA
PVL, mean (SD), log_10_ copies/mL	4.31 (1.28)	4.43 (1.11)	NA	NA	NA	0.303	NA
PVL <200 copies/mL, % (n)	12.4 (22/177)	6.1 (7/114)	NA	NA	NA	0.080	NA
HBV markers, % (n)							
All-negative	14.1 (28/198)	39.2 (188/479)	38.5 (145/377)	41.5 (90/217)	<0.001	<0.001	0.765
Isolated anti-HBc	6.1 (12/198)	9.6 (46/479)	6.9 (26/377)	5.1 (11/217)	0.127	0.134	0.087
Vaccination-type	55.1 (109/198)	35.7 (171/479)	41.9 (158/377)	40.6 (88/217)	<0.001	<0.001	0.153
HBsAg-positive	7.8 (18/232)	3.7 (19/507)	2.4 (9/377)	3.2 (7/217)	0.009	0.020	0.522
Anti-HBc-positive	30.3 (63/208)	26.3 (131/499)	19.6 (74/377)	18.0 (39/217)	0.003	0.273	0.015
Anti-HBs-positive	71.3 (154/216)	48.0 (245/510)	52.3 (197/377)	50.2 (109/217)	<0.001	<0.001	0.460
Anti-HBs titer, median, mIU/mL	96.55	63.25	NA	NA	NA	0.029	NA
<10 mIU/mL, % (n)	27.6 (58/210)	49.9 (225/451)	NA	NA	NA	<0.001	NA
>100	32.4 (68/210)	21.1 (95/451)	NA	NA	NA		
10–100	40.0 (84/210)	29.0 (131/451)	NA	NA	NA		
RPR >4, % (n)	19.8 (46/232)	21.2 (91/430)	2.8 (10/352)	0.5 (1/204)	<0.001	0.686	<0.001
Anti-HCV-positive, % (n)	4.3 (10/232)	3.5 (18/520)	1.1 (4/376)	1.4 (3/214)	0.031	0.570	0.045

a Comparison among the four groups.

b Comparison between Groups I and II.

c Comparison among the three groups born in or after 1986 (Groups II-IV).

**Abbreviations:** anti-HBc, hepatitis B virus core antibody; HBV, hepatitis B virus; HBsAg, HBV surface antigen; HCV, hepatitis C virus; RPR, rapid plasma reagin; PVL, plasma HIV RNA load; SD, standard deviation.

### Patterns of HBV serological markers for the four groups of patients

**Figure 1:**
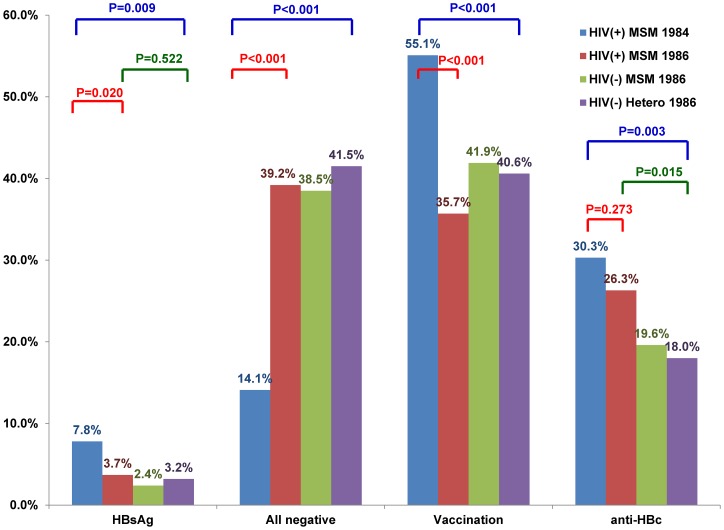
Comparisons of the proportions of persons with positive HBsAg (HBsAg), all-negative HBV markers (All-negative), vaccination serology (Vaccination), and positive anti-HBc (anti-HBc) among HIV-infected men who have sex with men (MSM) born during 1984–1985 (HIV[+] MSM 1984), HIV-infected MSM born in or after 1986 (HIV[+] MSM 1986), HIV-uninfected MSM and heterosexuals born in or after 1986 (HIV[−] MSM 1986 and (HIV[−] Hetero 1986).

The different serological patterns for the four study groups are shown in Table 1 and Figure 1. The proportions of patients with all-negative HBV markers were similar among Groups II-IV (39.2%, 38.5%, and 41.5%, respectively, *P* = 0.765), so were those of isolated anti-HBc (9.6%, 6.9%, and 5.1%, respectively, *P* = 0.087) and those of vaccination-type serology (35.7% vs. 41.9% and 40.6%, respectively, *P* = 0.153) (Table 1 and Figure 1). Compared with Group I, Group II were more likely to have all-negative HBV markers (39.2% vs. 14.1%, *P*<0.001) and less likely to have vaccination-type serology (35.7% vs. 55.1%, *P*<0.001), but had a similar proportion of isolated anti-HBc (9.6% vs. 6.1%, *P* = 0.134) (Table 1 and Figure 1); furthermore, Group I were significantly higher prevalence of HBsAg (7.8% vs. 3.7%, *P* = 0.020) and anti-HBs positivity (71.3% vs. 48.0%, *P*<0.001) than Group II, but both groups had a similar prevalence of anti-HBc positivity (30.3% vs. 26.3%, *P* = 0.273).

**Figure 2:**
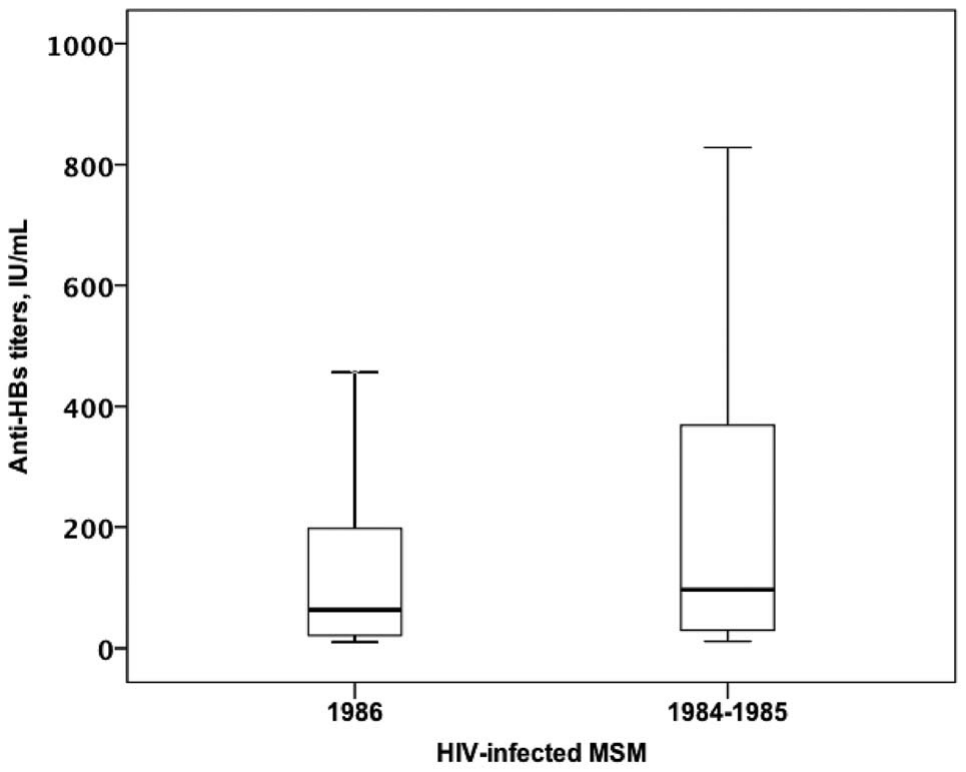
Boxplot of anti-HBs titers in HIV-infected MSM born during 1984-1985 and HIV-infected MSM born in or after 1986.

Among the 107 MSM in Group I and 154 MSM in Group II whose serological data of HBV and anti-HBs titers were available, MSM in Group I had a higher median anti-HBs antibody titer than those born in or after 1986 (96.55 vs. 63.25 mIU/mL, *P* = 0.029) (Figure 2).

### Syphilis and positive anti-HCV antibody

**Figure 3:**
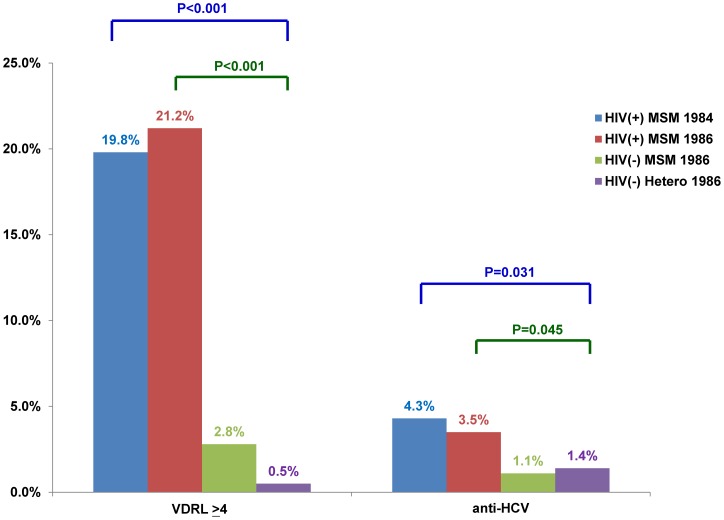
Comparisons of syphilis (RPR≧4) and anti-HCV positivity among HIV-infected MSM born during 1984–1985 (HIV[+] MSM 1984), HIV-infected MSM born in or after 1986 (HIV[+] MSM 1986), HIV-uninfected MSM and heterosexuals born in or after 1986 (HIV[−] MSM 1986 and (HIV[−] Hetero 1986).

Among the subjects in Groups II-IV, HIV-infected MSM in Group II had a higher prevalence of syphilis (21.2% vs. 2.8% and 0.5%, *P*<0.001) and anti-HCV positivity (3.5% vs. 1.1% and 1.4%, *P* = 0.031) than HIV-uninfected MSM in Group III and HIV-uninfected heterosexuals in Group IV, while similar prevalence of syphilis and anti-HCV positivity were observed between Groups I and II (syphilis, 19.8% vs. 21.2%; and anti-HCV positivity, 4.3% vs. 3.5%; both *P*>0.05) (Table 1 and Figure 3).

### Factors associated with positive HBsAg

Compared with the patients without positive HBsAg, those with positive HBsAg were older, and more likely to have HIV infection, to be born in 1984–1985, and to have syphilis and anti-HCV positivity than those without in univariate analysis (all *P*<0.05) (Table 2). In multivariate analysis (Table 3), HBsAg positivity was significantly associated with syphilis (odd ratios [OR], 2.990; 95% confidence interval [CI], 1.502–5.953, *P* = 0.002), and anti-HCV positivity (OR, 3.402; 95% CI, 1.091–10.614, *P* = 0.035), but not HIV infection (OR, 1.169; 95% CI, 0.531–2.577). Birth year in or after 1986 was associated with a lower risk for HBsAg positivity (OR, 0.421; 95% CI, 0.208–0.854, *P* = 0.017) (Table 3).

**Table 2:** Comparisons of study subjects with and without positive HBsAg in univariate analysis.

	Positive HBsAg	Negative HBsAg	*P* value
Patient no.	53 (4.0%)	1280 (96.0%)	
Age, mean (SD), years	26.8 (1.8)	25.7 (2.0)	<0.001
MSM, % (n)	84.8 (39/46)	82.5 (993/1203)	0.694
HIV infection	69.8 (37)	54.8 (702)	0.032
Born during 1984–1985	34.0 (18/53)	16.7 (214/1280)	0.001
Born in or after 1986	66.0 (35/53)	83.3 (1066/1280)	0.001
RPR titer >4	31.9 (15/47)	11.1 (128/1148)	<0.001
Positive anti-HCV	7.5 (4/53)	2.2 (28/1271)	0.03

**Abbreviations:** HCV, hepatitis C virus; MSM, men who have sex with men; RPR, rapid plasma reagin; SD, standard deviation.

**Table 3:** Multivariate analysis for factors associated with positive HBsAg.

Variables	Reference	Odds ratio	95% confidence interval	*P* value
HIV infection	No HIV infection	1.169	0.531–2.577	0.698
Born in or after 1986	Born during 1984–1985	0.421	0.208–0.854	0.017
RPR>4	RPR <4	2.990	1.502–5.953	0.002
Positive anti-HCV	Negative anti-HCV	3.402	1.091–10.614	0.035

### Incidence rate of natural HBV infection or chronic infection for HIV-infected MSM born in or after 1986

Among the 188 MSM in Group II who had all-negative HBV markers at baseline, 64 (34.0%) had follow-up of HBV serology for assessment of seroconversion for HBsAg, anti-HBs, anti-HBc as of 30 June, 2103. Compared with 124 MSM (66.0%) without follow-up of HBV serological markers, the 64 MSM had a similar age (24.8 [1.8] vs. 24.2 [2.1] years, *P* = 0.060) and prevalence of syphilis (13.1% vs. 16.9%, *P* = 0.503) and positive anti-HCV (0% vs. 0.8%, *P* = 0.999). Overall, one developed seroconversion of HBsAg (1.6%, 1/64), 18 patients anti-HBs positivity (28.1%, 18/64), and seven patients anti-HBc (10.9%, 7/64) at the end of follow-up (Table 4). The incidence rate of HBsAg seroconversion was 0.486 episodes per 100 PYFU, that of anti-HBs seroconversion was 9.897 episodes per 100 PYFU, and that of anti-HBc seroconversion was 3.578 episodes per 100 PYFU. The incidence rate of vaccination-type serological markers was 7.406 episodes per 100 PYFU, and serological markers consistent with natural HBV infection was 3.578 episodes per 100 PYFU (Table 4). Among the 64 patients, 49 (76.6%) patients subsequently underwent HBV vaccination during the follow-up, and only 13 patients (26.5%) developed vaccine-type serology.

**Table 4:** Crude incidence and incidence rate of seroconversion for HBsAg, anti-HBs or anti-HBc, HBsAg carriage, vaccination serology, and natural HBV infection.

	Crude incidence	Incidence rate (episodes per 100 person-years of follow-up)
All-negative HBV markers at baseline (n = 64), % (n)		
HBsAg seroconversion	1.6 (1/64)	0.486
Anti-HBs seroconversion	28.1 (18/64)	9.897
Anti-HBc seroconversion	10.9 (7/64)	3.576
Vaccine-type serology	21.9 (21/64)	7.406
Natural HBV infections	10.9 (7/64)	3.578
Receiving anti-HBV agents (n = 37), % (n)		
HBsAg seroconversion	0 (0/37)	0
Anti-HBs seroconversion	18.9 (7/37)	14.788
Anti-HBc seroconversion	8.1 (3/37)	6.338
Vaccine-type serology	13.5 (5/37)	10.563
Natural HBV infection	8.1 (3/37)	6.338

Among the 64 patients, 37 (57.8%) had follow-up of HBV markers when receiving combination antiretroviral therapy (cART) containing lamivudine or tenofovir disoproxil fumarate (TDF). During the treatment course for 47.334 PY, none developed HBsAg seroconversion, 7 (14.788 episodes per 100 PYFU) developed anti-HBs seroconversion, and 3 (6.338 episodes per 100 PYFU) developed anti-HBc seroconversion, 5 (10.563 episodes per 100 PYFU) developed vaccine-type serology, and 3 (6.338 episodes per 100 PYFU) developed HBV infection (Table 4).

## Discussion

In this study, we demonstrate that the prevalence of HBsAg positivity was similar between HIV-infected MSM and HIV-uninfected individuals (Group II-IV) who were born in the era of universal HBV vaccination (in or after 1986) despite the fact that HIV-infected MSM (Group II) were more likely to have syphilis and had a higher prevalence of anti-HBc positivity, while HIV-infected MSM born in 1984–1985 (Group I) had a significantly higher prevalence of HBsAg positivity than HIV-infected MSM in or after 1986 (Group II) (Table 1 and Figure 1). Syphilis and positive anti-HCV were significantly associated with HBsAg positivity (Table 3), suggesting sexual transmission may have contributed to HBV transmission in an area where mother-to-child transmission of HBV has become significantly less frequent after implementation of universal neonatal HBV vaccination program [24].

The latest seroepidemiologic survey in 2009 that was conducted 25 years after implementation of the universal HBV vaccination program to evaluate the effectiveness of program in the general population in Taiwan has shown that the prevalence of HBsAg positivity was 0.9%, and that of anti-HBs and anti-HBc positivity was 55.9% and 10.0%, respectively, in the subjects born in the vaccination era [25]. Among the subjects in the 1986–1989 birth cohort, the prevalence of HBsAg, anti-HBc, and anti-HBs was 1.6%, 0%, and 51.4%, respectively; and 44.9% of the subjects were negative for all three HBV serological markers [25].

In the four consecutive surveys conducted in 1994, 1999, 2004, and 2009, the prevalence of anti-HBc positivity, a surrogate marker of natural infection, increased gradually in the longitudinal follow-up of all birth cohorts, suggesting that exposure to HBV in the general population continued with age; however, HBsAg seropositivity did not increase among the cohorts [25], which suggests the long-term effectiveness in prevention against chronic HBV infection given that the risk of chronic HBV infection after exposure to HBV increases significantly with younger ages [26]. However, those surveys were conducted among the adolescents or young adults who had yet to become sexually active.

Compared with the findings of the survey in 2009 [25], all of our subjects in Groups II-IV had similar rate of anti-HBs positivity (48.0% to 52.3%), but a higher rate of anti-HBc positivity (18.0% to 26.3%) and lower rate of HBsAg positivity (2.4% to 3.7%) (Table 1). In addition, syphilis and positive anti-HCV were identified to be significantly associated with HBsAg positivity (Table 3). Our results suggest that the study subjects may have more opportunities of exposure to microbial agents that are sexually transmitted, such as HBV and syphilis, and might acquire HBV through high-risk sexual behaviors. Likewise, we have demonstrated HIV-infected patients with recent syphilis had a 7-fold greater risk for HCV seroconversion than those without [27]. Nevertheless, the prevalence of HBsAg positivity of HIV-infected MSM in Group II and HIV-uninfected MSM in Group III (3.7% and 2.4%) in the current study remains lower than that of HIV-infected and HIV-uninfected MSM who were born before 1986 (17.5% and 8.1%) in our previous survey [22]. Similarly, the prevalence of HBsAg positivity of HIV-infected patients born after July 1984 have declined significantly compared to those born before July 1984 (3.3% vs. 20.3%, *P*<0.05) [28], which declined further in those in Group II than in Group I (3.7% vs. 7.8%) in the present study (Table 1 and Figure 1), suggesting that universal HBV vaccination at birth and catch-up programs confer protection against HBsAg seroconversion among the adults engaged in high-risk sexual behaviors.

CART containing lamivudine or TDF has been recently shown to provide protection against HBV infection in HIV-infected Japanese [29]. The rate of incident HBV infection decreased from 6.726 per 100 PYFU in patients without cART to 0.669 per 100 PYFU in those on lamivudine or TDF-containing cART [29]. In the present study, the overall incidence rate of HBsAg seroconversion was 0.486 episodes per 100 PYFU in HIV-infected MSM born in or after 1986, and was 0 episodes per 100 PYFU in those receiving lamivudine or TDF (Table 4). Given the poor outcome of HBV infection in HIV-infected patients [14], early initiation of lamivudine or TDF-containing cART and adoption of safe sex practices are important for those who are sexually active and at risk for STDs but do not have protective levels of anti-HBs titers (<10 mIU/mL).

Revaccination is not recommended for vaccinated immunocompetent persons and adults with high-risk sexual behaviors [30], [31], but is recommended for immunocompromised hosts, such as patients undergoing continuous renal-replacement therapy [32], given the fact that clinically significant infections with HBV develop in those patients who have lost antibody for HBV [33]. However, whether HBV revaccination with regular follow-up of anti-HBs titer is indicated for HIV-infected patients who were born in vaccination era remains to be investigated.

There are several limitations to the present study. First, because of the retrospective study design, we were not able to document the HBV vaccination status and the date of vaccination at birth or revaccination of each person. However, given the high coverage rate of nationwide HBV vaccination program (86.9–98.0%) [20], and several catch-up programs of HBV vaccination to cover individuals born after 1977–1980 in their childhood [19], we believe that all of our study subjects who were born after 1986 in Taiwan had received HBV vaccination at birth or in the childhood. Second, whether maternal HBsAg carrier status or mutant viruses played a role in the HBsAg positivity in our study subjects remains unclear; these two factors might be related to vaccination failures [25], [34]. Nevertheless, given the findings that syphilis and positive anti-HCV were strongly associated with HBsAg positivity (Table 3), it is likely that HBV infection might have been acquired through sexual exposure. Last, we were not able to test HBV DNA in those with isolated anti-HBc, which might lead to underestimation of the prevalence or incidence of HBV infection since occult HBV infection may develop in patients with isolated anti-HBc [35].

In summary, the prevalence of HBsAg positivity has significantly declined in the adults engaged in high-risk sexual behaviors who were born in the era of nationwide HBV vaccination. While the prevalence of HBV infection remains low, safe sex counseling cannot be overemphasized because HBV infection is strongly associated with syphilis and positive anti-HCV.
